# Exceptional Strengthening Efficiency and Hardness of Ti/Mg-9Al-Zn-0.3Mn Matrix Composite

**DOI:** 10.3390/ma15207075

**Published:** 2022-10-11

**Authors:** Rongrong Wang, Yejin Han, Huan Yu, Qian Su, Hang Li, Kaiming Cheng, Jixue Zhou, Shouqiu Tang, Wei Ju

**Affiliations:** 1 Shandong Provincial Key Laboratory of High Strength Lightweight Metallic Materials, Advanced Materials Institute, Qilu University of Technology (Shandong Academy of Sciences), Jinan 250014, China; 2 School of Materials Science and Engineering, Shandong Jianzhu University, Jinan 250101, China

**Keywords:** metal-matrix composites, nano-crystalline, strengthening efficiency, interface, strengthening mechanisms

## Abstract

The involvement of magnesium matrix composite enhanced by metal particles, the development of low lattice mismatch interface, and the refining of particle size are all of great significance in improving strengthening efficiency. In this work, nano-crystalline Ti/Mg-9Al-Zn-0.3Mn composites were prepared by mechanical milling. The microstructure was characterized and the mechanical property was measured. After mechanical milling, the grain of the Mg matrix was refined to ~72 nm. Ti particles were smashed to submicron scale, and dispersed in the Mg matrix. In total, 68% of Ti particles were nano-scale and the average particle size was 133 nm. A nano-scale Mg_17_Al_12_ precipitate was found and the average particle size was approximately 44 nm. Meanwhile, coherent interfaces of Ti/Mg and Mg_17_Al_12_/Mg were observed, and it was found that the (101)Mg plane and (100)Ti plane inclined 12° and [044]Mg_17_Al_12_ was parallel to [010]Mg. The hardness of the milled Ti/Mg-9Al-Zn-0.3Mn composite was 1.98 GPa, 247% higher than the initial alloy. Milled Mg-9Al-Zn-0.3Mn alloy under the same preparation processing was used as a comparison, and the value of hardness was 1.53 GPa. Tiny Ti particles displayed excellent strengthening efficiency. Strengthening mechanisms of the milled Ti/Mg-9Al-Zn-0.3Mn composite were analyzed and the main strengthening mechanisms included the strengthening of grain boundary strengthening, Orowan strengthening, dislocation strengthening, solid solution strengthening and load-bearing strengthening, which accounted for 56.3%, 18.2%, 17.4%, 4.7% and 3.5%, respectively.

## 1. Introduction

Developing high-strength and lightweight materials is one of the most important issues in resolving the problems of the energy crisis and environmental pollution [1,2,3,4]. As the lightest metal structure material, magnesium alloy shows great potential in transport vehicle application and the aerospace industries due to its efficiency improvement in energy and carbon emission minimization [5]. However, serious drawbacks restrict further application—notably, insufficient strength, inferior ductility and poor thermal stability [6,7,8]. In order to overcome these shortcomings, ultrafine-grained (UFG) magnesium matrix composites (MMCs) have drawn growing interest [9,10,11,12,13].

Grain boundary (GB) strengthening is an effective strengthening mechanism for Mg matrix because it is particularly effective in hindering dislocation movement [6,14]. Sun W. et al. [15] obtained a nano-crystalline (NC) Mg-8.2Gd-3.8Y-1.0Zn-0.4Zr alloy with high hardness of 1.42 GPa by high-pressure torsion (HPT). NC Mg-Sm-Ca alloys with ultra-high hardness of ~1.72 GPa were prepared by hot rolling and HPT [16]. To improve the thermal stability of NC Mg matrix, discontinuous reinforcements such as SiC_p_ [13], Ti [17] and Mg_2_Cu [18] were usually introduced. It was found that the dispersion of submicron Ti (average size of 303 nm) can improve the thermal stability of NC AZ61, where the Mg matrix was still NC after heat treatment of 723 K for 600 min [19]. Su S. et al. [20] successfully prepared an NC AZ91 composite with ultra-high hardness of 1.81 GPa by dispersing submicron SiC_p_ and the average particle size was ~800 nm.

Unfortunately, the poor wettability and enormous difference in nature between ceramic and Mg matrix affect the bonding strength of the ceramic/Mg interface. Ceramic particles have been shown to damage the plasticity of MMCs [21,22]. Based on the above concerns, metallic particles were the preferred selection as discontinuous reinforcement of MMCs [23,24,25,26,27,28]. Zhang H. et al. [25] prepared a UFG V/AZ31 composite by powder metallurgy and the strength and ductility were improved due to submicron V particles (880 nm). Ti is also considered as ideal in particle reinforcement [26,29,30]. Since the crystal structures of both Ti and Mg are close-packed hexagonal, from the aspect of improving mechanical properties, a coherent interfacial bonding of Ti/Mg interface is expected. Previous studies [27,28,31] have confirmed the positive effect of Ti particles on mechanical properties. A firmly bonded Ti/Mg interface was observed. Li J. et al. [26] prepared a UFG Ti/Mg-3Al-Zn composite, where coherent interfacial bonding of the TiAl/MgO interface and semi-coherent interfacial bonding of the MgO/Mg interface formed. Compared with the unreinforced alloy, the elongation of 9Ti/Mg–3Al–1Zn was significantly increased by 81.8%. According to the Orowan strengthening mechanism [13,32], refining reinforcement would improve the strengthening efficiency. Chen L. et al. [32] achieved almost the same hardness as AZ91-15vol.%SiCp (submicron) [20] composite by only 6 vol.% nano-scale SiCp with semi-coherent bonding between SiC nano-particles and magnesium. This may also be applied to the MMCs enhanced by metal particles that form low lattice mismatch interfaces between particle and matrix more easily. However, as particle size decreases to below submicron level, the activity of metal particles increases sharply. The particle boundaries will adsorb or react with impurities, thus affecting the bonding interface with matrix. At present, the minimum particle size of Ti utilized as MMCs dispersion was achieved by the current authors for approximately 274 nm [33]. Accordingly, in order to advance the strengthening efficiency and obtain a composite with excellent performance, the low lattice mismatch interface and the increasingly smaller particle size were necessary to achieve.

In this work, Ti/Mg-9Al-Zn-0.3Mn composites were prepared by mechanical milling. The Ti/Mg interface was resolved by utilizing high-resolution transmission electron microscopy (HRTEM) characterization. The statistics of Ti particle size were investigated by utilizing scanning electron microscope (SEM) together with transmission electron microscopy (TEM) characterizations. Strengthening mechanisms were systematically analyzed according to the hardness results. The above theoretical work helps to facilitate the development of MMCs with high performance.

## 2. Experimental Procedure

### 2.1. Materials and Preparation

**Figure 1:**
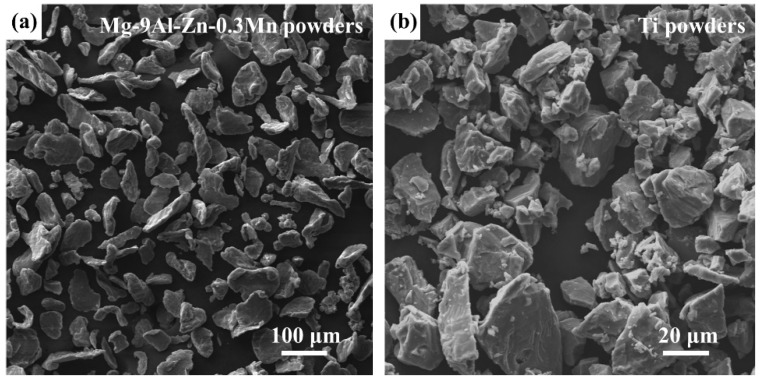
SE images of (**a**) Mg-9Al-Zn-0.3Mn powders and (**b**) Ti powders.

Powders of commercial pure Mg (99.5%, 325 mesh), pure Al (99.5%, 325 mesh), pure Zn (99%, 325 mesh) and pure Mn (99%, 325 mesh) were used as the raw materials and supplied by Jingding Alloy Material Co., Ltd. The nominal composition of mixed Mg alloy powders was Mg-9Al-Zn-0.3Mn in mass ratio. Pure Ti powders (99.9 mass%, 325 mesh) were used as the raw materials and supplied by Xi’an Baode Powder Metallurgy Co., Ltd. Figure 1 shows the secondary electron (SE) images of initially mixed Mg-9Al-Zn-0.3Mn powders and Ti powders.

Pre-crushing of Ti powders was performed with the milling rate of 200 rpm, milling time 16 h and ratio of milling balls to Ti powders of 30:1. Mechanical milling was applied to prepare Ti/Mg-9Al-Zn-0.3Mn composite powders with milling rate of 400 rpm, milling time of 12 h and the ratio of milling balls to composite powders of 20:1. In composite powders, the ratio of Ti phase was 10% in atom. To prevent agglomeration and excessive cold welding of powders, stearic acid was applied as process control agent and the weight ratio is 0.2%. The powders and the stainless steel milling balls were loaded into stainless steel vials under argon atmosphere (the purity superior to 99.99%) at ambient temperature. Powders were pressed to compacts with diameter of 15 mm and height of 10 mm under 1400 MPa at room temperature.

### 2.2. Microstructure and Properties Characterization 

SEM equipped with corresponding energy dispersive spectrometer (EDS) (EVOMA10) was applied to observe the microstructure. The X-ray diffraction (XRD, Bruker D8 advance) was performed on a D/max-*r*b diffractometer with Cu Kα radiation at a scanning rate of 2°/min. Before SEM and XRD tests, the testing surface of compacts was grinded, polished and cleaned in turn. Microhardness tester (HV, HV-1000) was applied with a load of 300 g and dwell time of 15 s. TEM (Talos F200X) was applied to observe the microstructures of milled alloy and composite. High angle annular dark field (HAADF), selected area electron diffraction (SAED) and HRTEM were utilized. Compact samples were mechanically ground to less than 50 μm, after that ion beam thinner (Gatan 691) was applied. Based on the Archimedes method, the density of materials was obtained by automatic density measuring instrument (JHY-300) with the accuracy of 0.001 g.

## 3. Results

### 3.1. XRD Results

**Figure 2:**
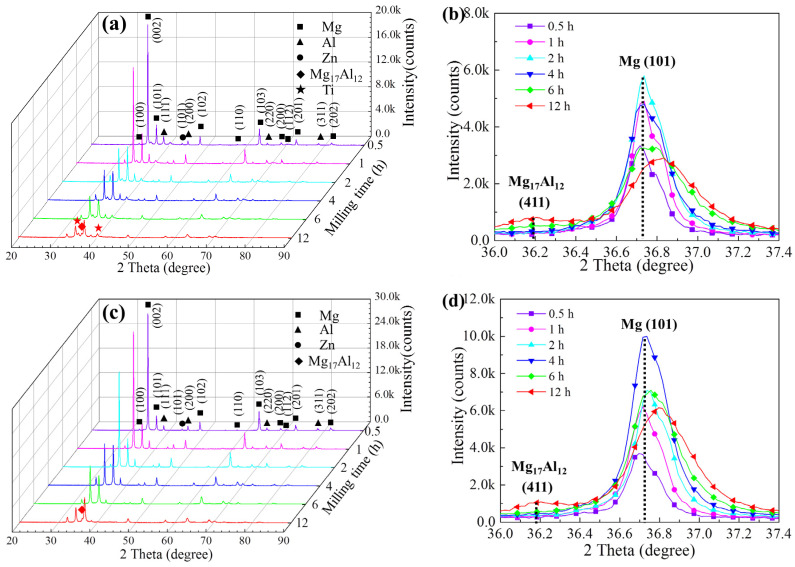
XRD patterns of the milled Ti/Mg-9Al-Zn-0.3Mn composite (**a**,**b**) and Mg-9Al-Zn-0.3Mn alloy (**c**,**d**) for various milling times.

XRD patterns of the milled Ti/Mg-9Al-Zn-0.3Mn composite and Mg-9Al-Zn-0.3Mn alloy for various milling times are shown in Figure 2. After milling for 0.5 h, diffraction peaks for Mg, Al, Zn and Ti phase can be found. With the increase in milling time, the diffraction peaks for Mg were widened considerably. The intensity for the (111) and (200) peaks of Al and the (101) peak of Zn decreased gradually. After milling for 12 h, the (200) peak of Al and (101) peak of Zn disappeared, and the weak (111) peak of Al was still observed. According to the peaks’ evolution, the Al and Zn phase were resolved during mechanical milling. Amplifying patterns (2 theta between 36.0°–37.4°) are shown in Figure 2b. The (411) peak of Mg_17_Al_12_ appeared after milling for 6 h and the intensity was increased after milling for 12 h. Thus, it can be confirmed that the Mg_17_Al_12_ phase was generated.

As shown in Figure 2c,d, the Mg_17_Al_12_ phase was also observed. The (200) peak of Al and (101) peak of Zn disappeared and the (411) peak of Mg_17_Al_12_ appeared after mechanical milling for 12 h, which was delayed in comparison with the Ti/Mg-9Al-Zn-0.3Mn composite. It can be inferred that Ti particles would accelerate atom diffusion or reaction and have little effect on the phase transformation during mechanical milling.

**Figure 3:**
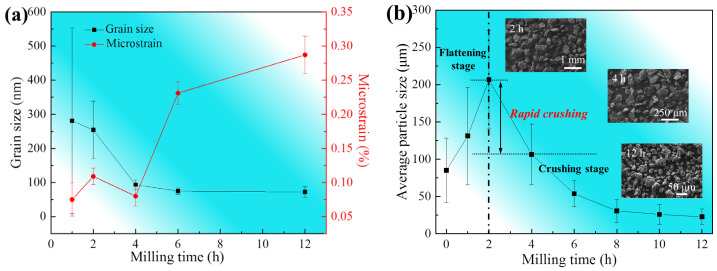
Evolution of grain size, the microstrain (**a**) and particle size (**b**) for the milled Ti/Mg-9Al-Zn-0.3Mn composite.

According to the XRD results, the evolution of the grain size and microstrain for Mg matrix is shown in Figure 3. The grain size and microstrain were calculated by the equations in a previous study [33]. The grain size of Mg matrix decreased continuously. After mechanical milling for 1 h, the average grain size was ~281 nm and the corresponding error bar was ~272 nm. During milling for 2 h to 4 h, grain size decreased considerably and the average grain size reached nano-scale with the value of 94 nm. The refinement of grain can be attributed to the piling up of dislocations. With the decreasing of grain size, the content of GB increased at a speed of the index. Dislocations were annihilated at GBs more easily and further grain refining required more energy input. Thus, the grain size tended to be stable. After mechanical milling for 12 h, the average grain size was ~72 nm.

With an increase in milling, the microstrain increased gradually. However, during milling for 2 h to 4 h, the microstrain decreased abnormally. The above phenomenon could attribute to rapid crushing of particles shown by Figure 3b, which released part of the microstrain. After mechanical milling for 4 h, the Ti particles dispersed well in Mg matrix, which led to the restriction of GB movement and grain rotation. During this period, dislocation evolution including multiplication and movement accelerated and the microstrain increased rapidly.

According to the XRD results, dislocation density ρ can be calculated by the following equation [34,35]:

(1) $$\rho =\frac{2\sqrt{3}{\langle \varepsilon^{2}\rangle }^{1/2}}{db}$$

where ε is the microstrain, b is the Burgers vector (0.3197 nm) and d is the grain size. Thus the ρ values of milled Ti/Mg-9Al-Zn-0.3Mn composite for 12 h and Mg-9Al-Zn-0.3Mn alloy were provided by Table 1.

### 3.2. Microstructure Characterization

**Figure 4:**
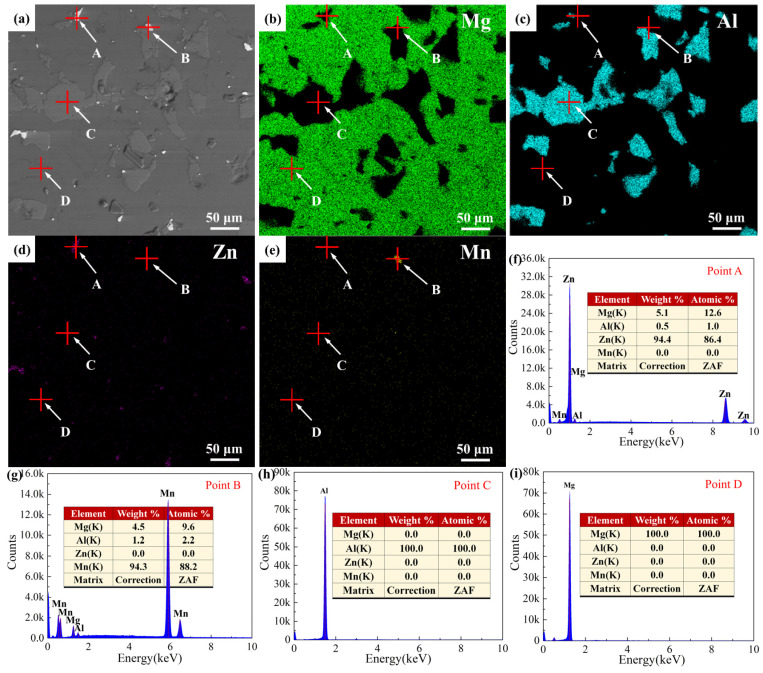
Microstructure of Mg-9Al-Zn-0.3Mn: (**a**) BSE image; (**b**–**e**) element distribution results; (**f**–**i**) EDS results of points A–D in (**a**).

The microstructure of the initial Mg-9Al-Zn-0.3Mn powder is shown in Figure 4. The mixed powder was composed by Mg, Al, Zn and Mn phase and no diffusion was observed, based on the back-scattered electron (BSE) images and the EDS results. The Al phase was distributed uniformly in Mg matrix.

**Figure 5:**
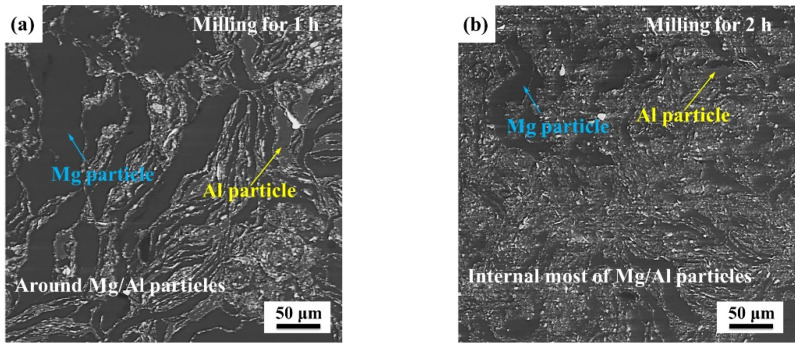

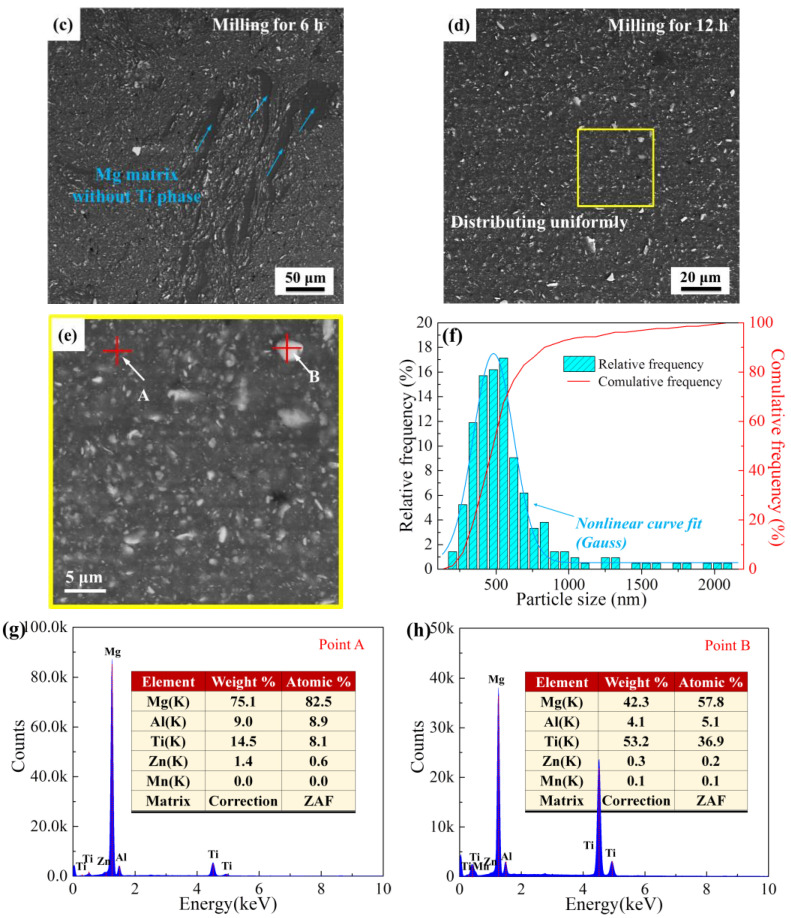
BSE images of milled composite powers for various milling times: (**a**) 1 h; (**b**) 2 h; (**c**) 4 h and (**d**,**e**) 12 h, (**f**) particle size statistics of (**e**) and (**g**,**h**) EDS results of A and B in (**e**).

During mechanical milling, the mixed powders exhibited plastic deformation under the collision effect from high-speed balls. The microstructure of the milled composite powers for various milling times is shown in Figure 5. At the initial stage, Ti particles encased the Mg and Al phase as shown in Figure 5a. Cold welding of the ductile Mg and Al powders surrounded by the brittle Ti phase would be prevented. Additionally, when the defect rooting in the dislocation pile-up reached critical value, ductile particles were crushed to fragments. With the Ti phase entered into the ductile particles, shown by Figure 5b,c, the brittle Ti phase surrounding the ductile particle was insufficient to prevent cold welding. Additionally, under the effect cold welding and crushing, the Ti phase was distributed uniformly in Mg matrix as shown by Figure 5d.

An amplified SEM image of the yellow box area in Figure 5d is shown in Figure 5e. EDS results of point A and B are shown in Figure 5g,h. Accordingly, the Ti element agglomerated in the B particle region. Meanwhile, the Ti phase was found in XRD patterns as shown by Figure 2. Thus, the particle region identified as B in Figure 5e was the Ti phase. Additionally, the gray region was Mg matrix. No hole was observed in Figure 5d,e. The physical density of the Mg-9Al-Zn-0.3Mn alloy was 1.83 g/cm^3^. According to the rule of mixtures, the physical density of the Ti/Mg-9Al-Zn-0.3Mn composite was 2.00 g/cm^3^. The testing density of the pressed composite was approximately 98.5% and the porosity was 1.5%. Thus, after the pressing process, the Ti/Mg-9Al-Zn-0.3Mn composite was almost densified. The results of the particle size statistics in Figure 5e were provided by Figure 5f. The maximum size of the Ti particles was approximately 3.2 μm and the minimum value was approximately 230 nm. Size distribution of the Ti particles coincided with the Gauss model. There were approximately 93% Ti particles reaching submicron scale.

**Figure 6:**
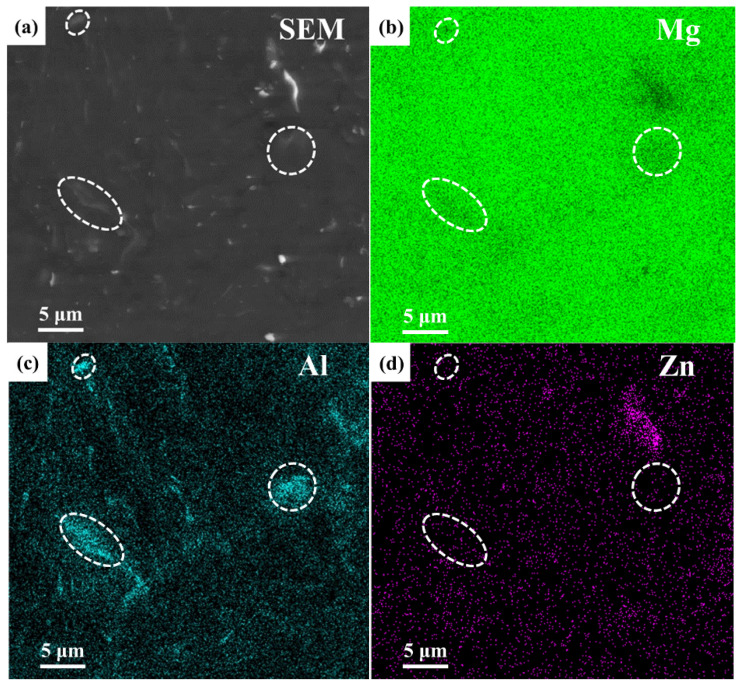
The microstructure milled Mg-9Al-Zn-0.3Mn alloy: (**a**) BSE image, (**b**–**d**) element distribution results.

The microstructure of the milled Mg-9Al-Zn-0.3Mn alloy is shown by Figure 6. After mechanical milling, the Al element dissolved into Mg matrix. Additionally, there was still segregation of the Al element marked by white ellipses. In comparison with the milled composite shown by Figure 5, the residual Al phase of the milled alloy was much higher. The same conclusion can also be drawn by the evolution of the (200) and (220) peaks for the Al phase during mechanical milling as shown by Figure 3. During mechanical milling, the addition of Ti particles increased the microstrain by restricting grain movement and accelerated the Mg grain refining process. The crystal defects of the composite such as dislocation and grain boundary increased, which extended atom diffusion channels. Thus, the Al phase dissolving into Mg matrix became more active.

**Figure 7:**
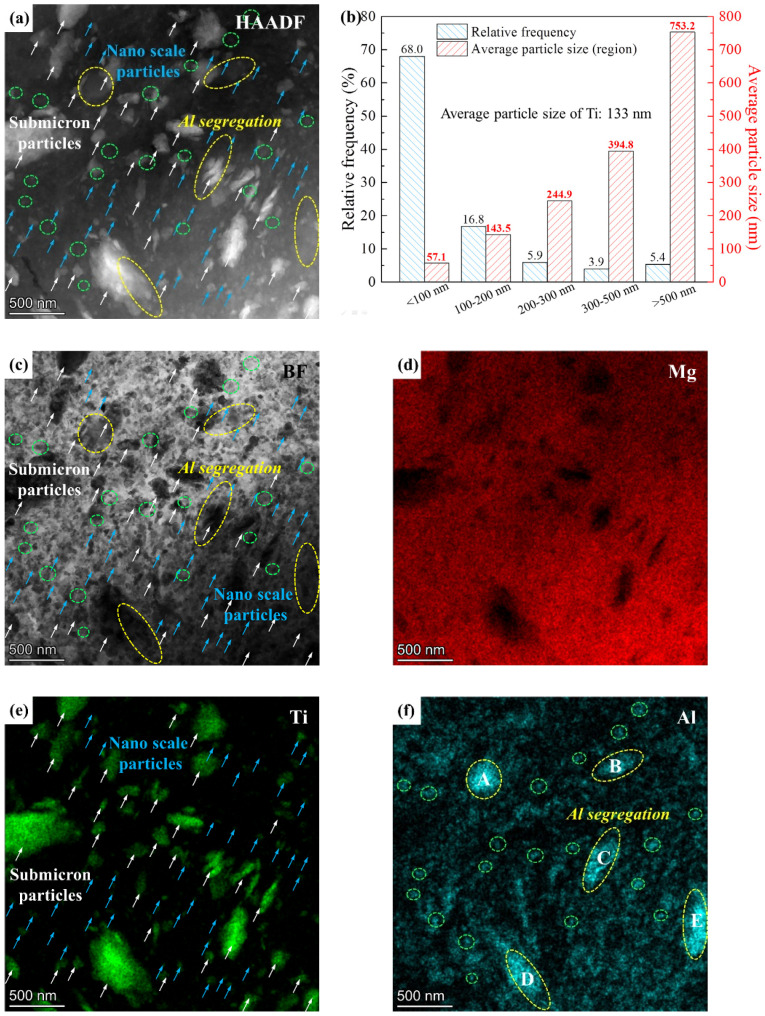
Microstructure of the milled Ti/Mg-9Al-Zn-0.3Mn composite: (**a**) HAADF image, (**b**) particle size statistics of (**a**,**c**) BF image, (**d**–**f**) element distribution results.

To characterize nano-scale Ti particles, a TEM test of milled Ti/Mg-9Al-Zn-0.3Mn composite was performed. HAADF image, BF image and corresponding element distribution are shown in Figure 7. As shown by Figure 7a,e, the white particles were Ti phase. Submicron particles are indicated by white arrows and nano-scale particles are pointed out by blue arrows. The results of the particle size statistics in Figure 7a are shown in Figure 7b. Most of the Ti particles were smaller than 100 nm. The Ti particles are marked in the BF image shown by Figure 7c, and the Ti particles are distributed uniformly in NC Mg matrix. Combining the results of SEM and TEM, the statistics of relative frequency and corresponding average particle size for different particle size regions are shown in Figure 7b. In total, 68% of Ti particles with an average size of 57.1 nm were nano-scale. The average particle size $S_{0}$ can be calculated by the formula: $S_{0}=\sum S_{i}f_{i}$, where $S_{i}$ and $f_{i}$ were average particle size of relative frequency for different size region. Thus, the average particle size was calculated to be ~133 nm.

As shown by Figure 7f, segregation of the Al element was observed. The area of Al segregation was submicron scale marked by yellow ellipses, and nano-scale marked by purple ellipses. According to the HAADF and bright field (BF) images, submicron segregation area of the Al element falls broadly into two categories: Al phase marked by A, B and E in Figure 7f and Al element dissolved in the Ti phase marked by C and D in Figure 7f. The nano-scale segregation area may be attributed to the NC Mg_17_Al_12_ precipitate or Al phase. In addition to the segregation mentioned above, it can be confirmed that the Al element dissolved into the Mg matrix.

**Figure 8:**
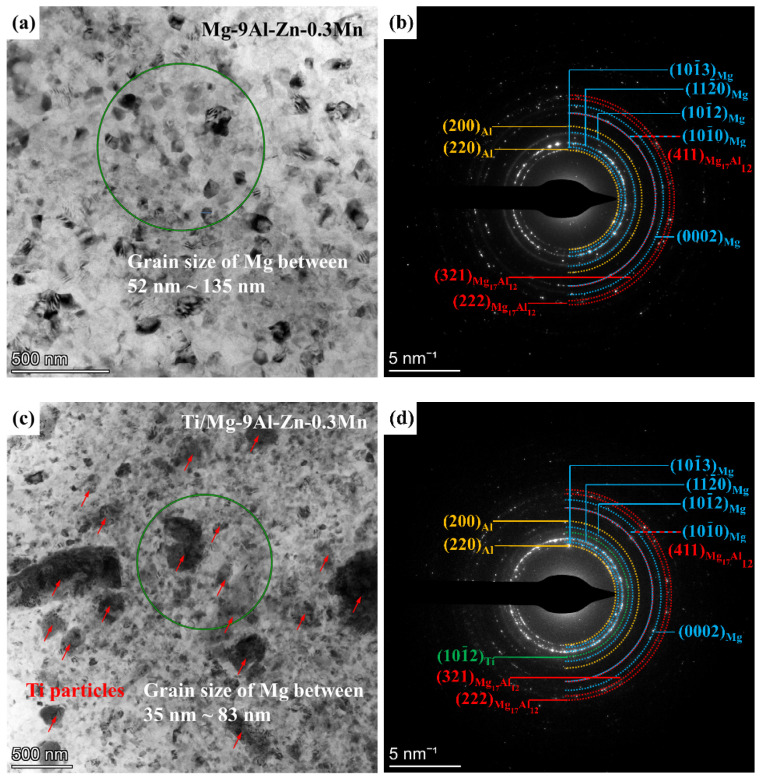
BF images and corresponding SAED results of milled Mg-9Al-Zn-0.3Mn (**a**,**b**) and Ti/Mg-9Al-Zn-0.3Mn (**c**,**d**).

BF and corresponding SAED images of the milled Mg-9Al-Zn-0.3Mn alloy and Ti/Mg-9Al-Zn-0.3Mn composite are shown in Figure 8. As shown by Figure 8a, the grain size of Mg for the milled alloy was between 52 nm and 135 nm. According to the SAED results of the green circle area in Figure 8a, the milled alloy was composed of Mg, Al and Mg_17_Al_12_. Additionally, the grain size would be submicron scale. The grain size of the Mg matrix for the milled composite was between 35 nm and 83 nm. The Ti particles are marked by red arrows. Additionally, the Ti phase (1012) was found. After the same mechanical milling processing, the smaller grain size of the composite compared to the Mg alloy should be attributed to the dispersing Ti particles. During mechanical milling, Mg matrix exhibited plastic deformation rooting in dislocation evolution, grain boundaries movement and grain rotation. As shown by Figure 5, the Ti particles dispersed in most of the matrix after 4 h milling, which reduced the opportunity for grain boundary movement and grain rotation for the Mg matrix. Thus, the generation and multiplication of dislocation were enhanced, which accelerated grain refining.

**Figure 9:**
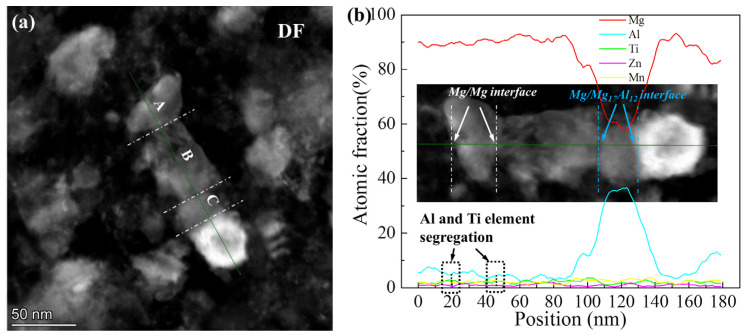
DF image (**a**) and corresponding element content results (**b**) of the milled Ti/Mg-9Al-Zn-0.3Mn composite.

The dark field (DF) image and corresponding element content results are shown in Figure 9. According to the atomic fraction of the line in Figure 9a, the A and B areas were Mg phase and the C area was Mg_17_Al_12_ phase. The crystalline sizes of both Mg and Mg_17_Al_12_ were nano-scale. It was confirmed that the Al and Ti elements segregated at GBs due to the higher distortion energy of GB atoms. Similar results were confirmed in a previous study [30]. The solid solubility of Al and Ti in the Mg matrix was estimated by the atomic fraction results of area A and the values are 4.7 at.% and 2.2 at.%, respectively.

### 3.3. Mechanical Properties

**Figure 10:**
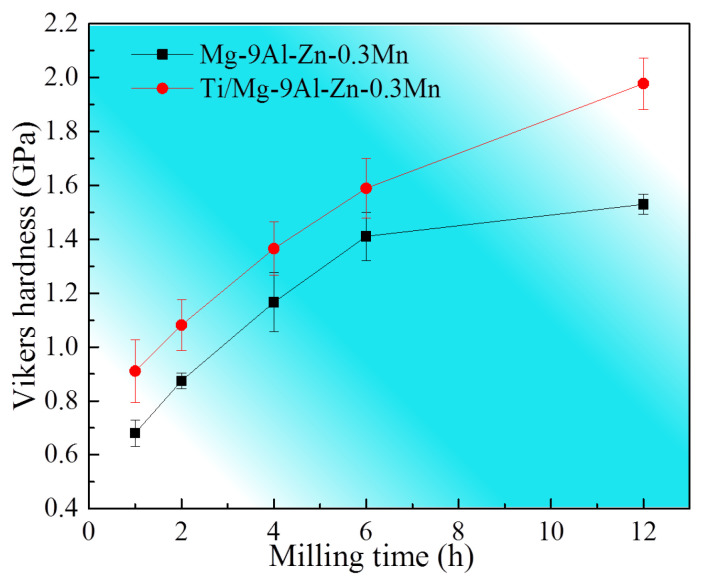
Hardness evolution of the milled Mg-9Al-Zn-0.3Mn alloy and Ti/Mg-9Al-Zn-0.3Mn composite.

The hardness evolution of the milled Mg-9Al-Zn-0.3Mn alloy and Ti/Mg-9Al-Zn-0.3Mn composite for various milling times is shown by Figure 10. The hardness of the initial Mg-9Al-Zn-0.3Mn was 0.57 GPa. With the increase in milling time, the harness increased considerably. Additionally, during the same milling time, the hardness of the composite was higher than that value of the alloy, which can be attributed to the Ti particles and a finer grain of the Mg matrix. After mechanical milling for 12 h, hardness values of the alloy and composite reached 1.53 GPa and 1.98 GPa with increasing ratios of 168% and 247%, respectively.

## 4. Discussion

### 4.1. Interfacial Bonding Analysis

**Figure 11:**
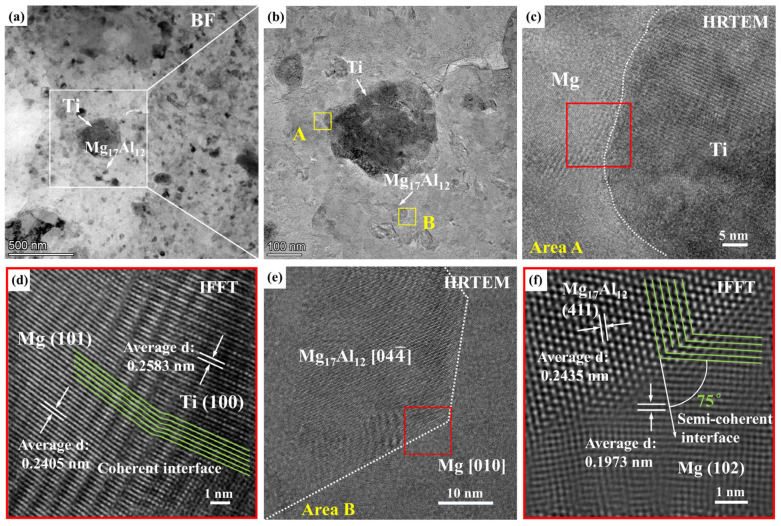
Microstructure of the milled Ti/Mg-9Al-Zn-0.3Mn composite: (**a**) BF image, (**b**) large view HRTEM of white square in (**a**,**c**,**e**) HRTEM of areas A and B in (**b**) characterizing Ti/Mg and Mg_17_Al_12_/Mg interface, (**d**,**f**) IEEF image of square regions.

As mentioned above, submicron Ti particles and Mg_17_Al_12_ precipitates dispersed in the Mg matrix. The interface condition of Ti/Mg and Mg_17_Al_12_/Mg was characterized by Figure 11. The HRTEM image of the white square area in Figure 11a is shown in Figure 11b. According to the HRTEM results, Ti particles with size of ~300 nm and Mg_17_Al_12_ precipitate with size of ~44 nm are marked by white arrows. The size of the Mg_17_Al_12_ precipitate matched well with the nano-scale segregation of the Al element shown by Figure 7. Inverse fast Fourier transform (IFFT) image of the red area in Figure 11c for Ti/Mg interface is shown by Figure 11d. As shown, the orientation relationship of the (101)Mg plane and (100)Ti plane inclined at 12° and a coherent interface was confirmed. Mechanical milling processing crushed the Ti particles to a submicron scale and plenty of fresh Ti particle interfaces formed. Moreover, both Mg and Ti crystallize in close-packed hexagonal structures. Displacement between Ti particles and the Mg matrix occurred under the effect of collision. The near-atomic structure would accelerate atomic inter-diffusion, which caused coherent interface generation easily. Thus, a good combination of Mg and Ti phase was anticipated. The interface between the matrix and the reinforcements plays a key part in the development of high-performance nano-composites. A coherent interface between Ti particles and the Mg matrix was observed, which should result in strong interfacial bonding. Thus, Ti particles can bear the load transmitted by the metal matrix well and the probability of crack generation at the Mg/Ti interface would decrease.

The HRTEM image of area B in Figure 11b is shown in Figure 11e. Both [044] zone axis of Mg_17_Al_12_ precipitate and [010] zone axis of Mg matrix were parallel to the electron beam. That means [044]Mg_17_Al_12_//[010]Mg. The corresponding IFFT image of the red square in Figure 11e is shown by Figure 11f. It revealed that (411)Mg_17_Al_12_ and (102)Mg present an intersection angle of 75° and the Mg_17_Al_12_/Mg interface was confirmed to be a coherent interface. The forming mechanism of the Mg_17_Al_12_ phase differed from the solidification processing. The Al phase was crushed to nano-scale particles, during which the Mg element diffused and then reacted with Al. The superior diffusion channel was selected spontaneously. Thus, the Mg_17_Al_12_/Mg coherent interface became the priority. The low lattice misfit between the second phase and matrix would improve the mechanical properties of the composite [16].

### 4.2. Strengthening Efficiency Analysis

**Figure 12:**
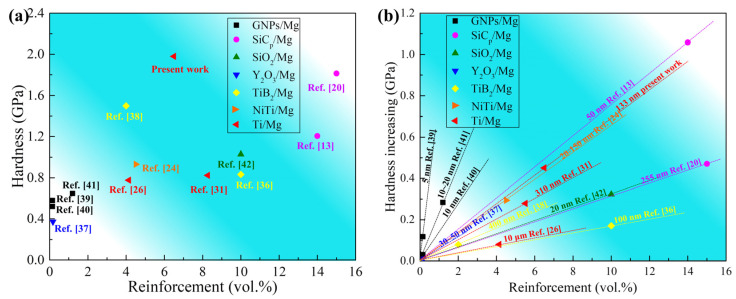
Hardness (**a**) and increasing harness (**b**) of MMCs reinforced by various particles [13,20,24,26,31,36,37,38,39,40,41,42].

The hardness and increasing harness of MMCs reinforced by various particles are collected and shown in Figure 12 [13,20,24,26,31,36,37,38,39,40,41,42]. The Ti/Mg-9Al-Zn-0.3Mn composite in the present work was of ultra-high hardness. Figure 12b presents the relationship of increasing hardness and reinforcement content. In this work, the enhancing efficiency (*E*) of different particles was quantified by the slope value. The typical size including thickness for graphene nano-particles (GNPs) and diameter for particles was also provided. As shown in Figure 11b, GNPs possessed outstanding efficiency and the values of $E_{GNPs}$ were 78.4, 23.7 and 19.6, respectively, which can be attributed to the small thickness of less than 20 nm. Additionally, the GNPs/Mg composite with the smallest thickness has the highest strengthening efficiency. However, due to the nature of GNPs, the increasing content would not bring about the continued improving of strength [39], which blocked further research on GNPs/Mg composites. It can be confirmed that the slopes for composites were related to particle size. Usually, refining the particle size would improve the strengthening efficiency for the same reinforcement such as GNPs, SiC_p_ and Ti shown in Figure 12b. For TiB_2_ reinforcement, the opposite trend was found. The slope value of the composite enhanced by TiB_2_ with a particle size of ~100 nm was less than the value of that composite with a particle size of ~400 nm [9,36]. In comparison with Ti distribution, it can be inferred that dispersing was necessary to realize the ideal strengthening efficiency.

$E_{{SiC}_{p}}$ of the Mg2Zn composite enhanced by SiC_p_ with a size of 50 nm was highest in MMSc enhanced by particles except for GNPs, and the slope value was 7.6. The outstanding result can be attributed to the special preparation process. Dispersing nano-scale SiC_p_ uniformly and achieving good bonding between SiC_p_ and the Mg matrix were realized [13]. For metal or alloy reinforcement, interface bonding was the superior method to improve the strengthening efficiency in comparison to ceramics. For the NiTi/Mg–3Zn-0.5Ag composite [24], the size of the NiTi particles was between 20–150 nm and the slope value was 6.5. In the present work, the slope value of the Ti/Mg-9Al-Zn-0.3Mn composite was 6.9, near the $E_{{SiC}_{p}}$ of SiC_p_/Mg2Zn. The coherent interface of Ti/Mg shown in Figure 11 provided stronger interfacial bonding, which was the source of excellent strengthening efficiency. In addition, the average size of tiny Ti particles was 133 nm and 68% of those were nano-scale, promoting the further improvement of strengthening efficiency.

### 4.3. Strengthening Mechanisms

**Figure 13:**
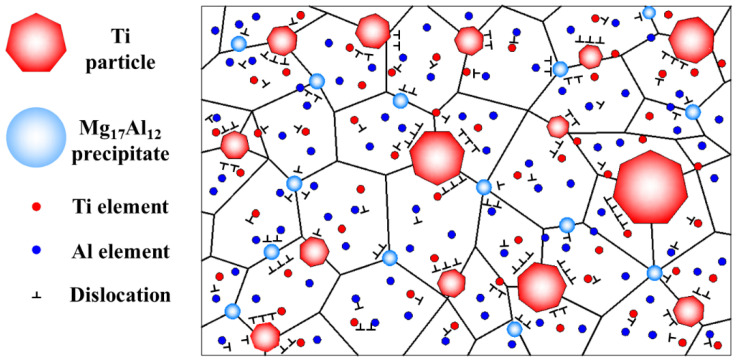
The schematic illustration of microstructure for the milled Ti/Mg-9Al-Zn-0.3Mn composite.

According to the relationship between hardness and yield strength [43], the yield strength of the milled composite and alloy was calculated to be 673 MPa and 520 MPa, respectively. In comparison with initial pure Mg (147 MPa), the increasing values were 526 MPa and 373 MPa, respectively. The improved strength was attributed to the microstructure of dislocations, grain boundaries (GBs), solute atoms, and second-phase particles. The schematic illustration of the microstructure for the milled Ti/Mg-9Al-Zn-0.3Mn composite is shown by Figure 13. In this section, a model for hardening was constructed and strengthening mechanisms were analyzed from [13,31,44]: dislocation strengthening ($\Delta \sigma_{DS}$), GBs strengthening ($\Delta \sigma_{GB}$), solid solution strengthening ($\Delta \sigma_{SS}$), load-bearing strengthening ($\Delta \sigma_{LB}$) and Orowan strengthening ($\Delta \sigma_{OR}$).

During mechanical milling, dislocation evolution, including generating, movement and pile-up, induced an increase in dislocation density and grain refining of the matrix.

For dislocation strengthening, the increasing strength $\Delta \sigma_{DS}$ was influenced by dislocation density (ρ) and calculated as follows [44,45]:

(2) $$\Delta \sigma_{DS}=\tau MGb\sqrt{\rho }$$

where τ is the Taylor factor (taken as 2.6), M is a constant (approximately 0.3), G is the shear modulus (17.7 GPa), ρ was measured as $4.3\times 10^{14}$ m^−2^ and $2.3\times 10^{14}$ m^−2^ for the composite and alloy. Thus, the corresponding $\Delta \sigma_{DS}$ values were calculated as 92 MPa and 67 MPa.

Grain boundaries cannot be penetrated by a moving dislocation. Generally, Hall–Petch relation was applied and the increasing strength was calculated using the following formula [46]:

(3) $$\Delta \sigma_{GB}=kd^{-1/2}$$

where k is the Hall–Petch constant. k is an empirical value and varied with grain size. Li X. et al. [13] prepared an NC Mg2Zn alloy (105 nm) and SiC/Mg2Zn composite (64 nm). The increment in strength was calculated as 280 MPa and 300 MPa, respectively. An NC composite (72 nm) and alloy (91 nm) were obtained. Therefore, according to the above results, the $\Delta \sigma_{GB}$ values for the milled composite and alloy were calculated as 296 MPa and 287 MPa, respectively.

Mechanical milling resulted in the dissolving of solute atoms and then lattice distortion was triggered. For solid solution strengthening, the increment $\Delta \sigma_{SS}$ can by estimated as follows [47]:

(4) $$\Delta \sigma_{SS}=3^{3/2}\frac{G\delta^{3/2}c^{1/2}}{700}$$

where δ is mismatch parameter and c is the atomic fraction of the solute. δ for Al/Mg and Ti/Mg was 0.31 and 1.06, respectively [31]. According to the EDS line results shown in Figure 9, compositions of Al and Ti in the Mg matrix were 4.7 at.% and 2.2 at.%, respectively. Additionally, the $\Delta \sigma_{SS}$ values for composite and alloy were calculated as 25 MPa and 5 MPa, respectively.

The formation of a Ti supersaturated Mg solid solution was confirmed. With the same initial Ti phase content and lower milling intensity, the solid solubility of Ti in Mg was higher than the value of a previous study (1.1 at.%) [33]. The results would account for the increasing Ti/Mg interface atoms. The average particle size of Ti dispersion in the present study (133 nm) was smaller than the value (274 nm) of the previous study. Thus, the atom quantity was increased by approximately 2.1 times, which provides more channels for diffusion. Zhou H. et al. [48] prepared Ti/Mg composite powders by mechanical milling. It was concluded that the solid solubility of Ti in Mg was related to the quantity of the Mg/Ti interface atoms.

For dislocation strengthening, the increasing strength was estimated by the following formula [49]:

(5) $$\Delta \sigma_{LB}=0.5V_{p}\sigma_{m}$$

where $V_{p}$ is the volume fraction of particles and $\sigma_{m}$ is the yield strength of the matrix. $\sigma_{m}$ was the sum of yield strength of pure Mg ($\sigma_{0}$), $\Delta \sigma_{GB}$ and $\Delta \sigma_{SS}$ for Al dissolving in the Mg matrix. Thus the $\Delta \sigma_{LB}$ value for the composite was calculated as 18 MPa.

The contribution by the Orowan strengthening mechanism induced by well dispersed particles and $\Delta \sigma_{OR}$ can be calculated by the following equation [13]:

(6) $$\Delta \sigma_{OR}=\frac{\phi Gb}{d_{p}}{\left(\frac{6V_{p}}{\pi }\right)}^{1/3}$$

where ϕ is a constant (equal to 2) and $d_{p}$ is the size of the dispersions. After mechanical milling, the average particle size of Ti was approximately 133 nm. Thus, the $\Delta \sigma_{OR}$ value due to Ti particles for the composite was calculated as 42 MPa. Nano-scale Mg_17_Al_12_ precipitates also enhanced the Mg matrix. However, due to some of the Al element dissolving in Ti particles or existing as Al phase shown by Figure 7, the volume fraction of the precipitates was not available. Hence, the residual contribution to strength was attributed to the precipitates. Therefore, the $\Delta \sigma_{OR}$ values due to the Mg_17_Al_12_ precipitates for the composite and alloy were calculated as 53 MPa and 14 MPa, respectively.

**Figure 14:**
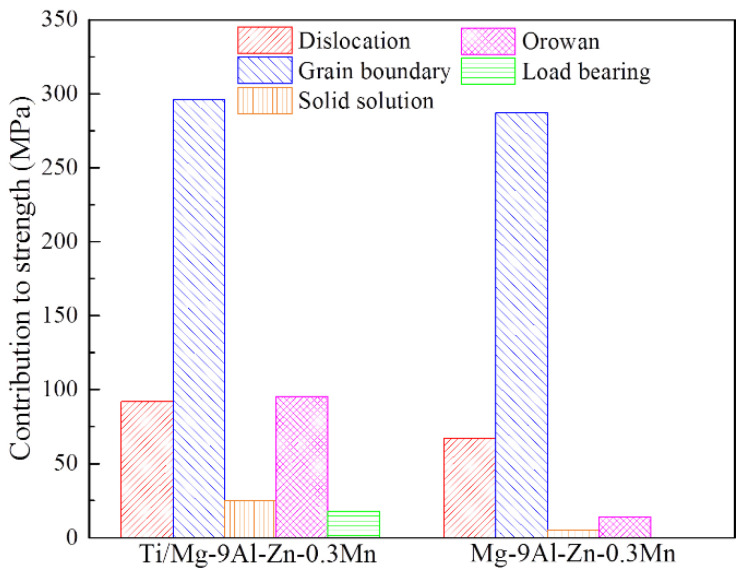
The contribution to strength from different strengthening mechanisms.

The contribution of the above strengthening mechanisms is shown by Figure 14. For the composite, the main strengthening was attributed to GBs strengthening, dislocation strengthening and Orowan strengthening, which accounted for 56%, 17% and 18%, respectively. Additionally, in comparison with alloy, the improved strength was approximately 152 MPa. During mechanical milling, the dispersing Ti particles altered the dislocation evolution, refined the Mg grain and accelerated the resolving Al phase, which resulted in higher dislocation density, finer Mg grain and more nano-scale precipitates. Furthermore, the Ti element became a supersaturated solid solution in the Mg matrix. The contributions of various strengthening mechanisms to enhanced strength were Orowan strengthening (81 MPa), dislocation strengthening (24 MPa), solid solution strengthening (20 MPa), load-bearing strengthening (18 MPa), and GBs strengthening (9 MPa).

## 5. Conclusions

In this study, NC Ti/Mg-9Al-Zn-0.3Mn composites were prepared by mechanical milling. The microstructure of the milled composite was characterized and its strengthening mechanisms were analyzed. The main conclusions can be summarized as follows:

(1) The NC Ti/Mg-9Al-Zn-0.3Mn composite was obtained. Submicron Ti particles and nano-scale Mg_17_Al_12_ precipitates were dispersed in the Mg matrix. A total of 68% of Ti particles were refined to nano-scale. The average grain size of the Mg matrix was ~72 nm. A Ti supersaturated Mg solid solution formed, and the solid solubility was 2.2 at.%.
(2) The ultra-high hardness of MMCS was achieved. The hardness of the milled Mg-9Al-Zn-0.3Mn alloy and Ti/Mg-9Al-Zn-0.3Mn composite was 1.53 GPa and 1.98 GPa, respectively, 168% and 247% higher than the initial alloy. The tiny Ti particle was of excellent strengthening efficiency. The additional improvement of the composite was attributed to higher dislocation density, finer Mg grain and more nano-scale precipitates caused by submicron Ti dispersions.
(3) A strong interfacial bonding was formed between the Mg matrix and second phase. A coherent interface of Ti/Mg was confirmed and the orientation relationship of the (101)Mg plane and (100)Ti plane inclined at 12°. A coherent interface between nano-scale Mg_17_Al_12_ and the Mg matrix was observed and the orientation relationship [044]Mg_17_Al_12_//[010]Mg was certified.
(4) The strengthening mechanisms were systematically analyzed. Strengthening mechanisms were attributed to GBs strengthening, Orowan strengthening, dislocation strengthening, solid solution strengthening and load-bearing strengthening, accounting for 56.3%, 18.2%, 17.4%, 4.7% and 3.5%, respectively.

## Figures and Tables

**Table 1:** The grain size, microstrain, dislocation density of the milled composite and alloy.

Sample	Grain Size *d* (nm)	Microstrain *ε* (%)	Dislocation Density *ρ* (×10^14^ m^−2^)
Ti/Mg-9Al-Zn-0.3Mn composite	72.0	0.287	4.3
Mg-9Al-Zn-0.3Mn alloy	91.1	0.196	2.3

## Data Availability

All data are available from the corresponding author on reasonable request.
